# Catalytic Strategies for Stereoselective Carbohydrate Synthesis: Emerging Concepts for Accessing Challenging Glycosides

**DOI:** 10.1002/anie.202514167

**Published:** 2025-09-13

**Authors:** Charles C. J. Loh

**Affiliations:** ^1^ UCD School of Chemistry University College Dublin Belfield, Dublin 4 Ireland

**Keywords:** Carbohydrates, Catalysis, Glycosylation, Noncovalent interactions, Stereoselectivity

## Abstract

The development of innovative catalytic strategies for stereoselective carbohydrate synthesis is rapidly emerging as a theme of general synthetic interest. This research frontier is fuelled by current realization that anomeric and site‐selectivity challenges encountered in carbohydrate chemistry are indeed fascinating fundamental synthetic questions, still yet to be satisfactorily addressed. Although specialist carbohydrate chemistry and catalysis developed largely independently over the past three decades, growing recognition of carbohydrates as an excellent platform for catalytic method development is now generating fresh opportunities to advance our understanding of chemical phenomena. Unexpected catalytic mechanisms are often unearthed in such cross‐disciplinary endeavours. Moreover, new bond‐forming catalytic solutions are in great demand as the definition of biologically relevant glycosidic chemical space has broadened substantially beyond native oligosaccharides to include glycomimetics, rare and non‐classical sugars. Distinctly, the last 5 years is marked by an unprecedented flurry of activity in the development of ground‐breaking catalytic carbohydrate transformations, leading to robust methods that diverge from classical glycosylation approaches. In this minireview, lately emerging concepts such as noncovalent catalyzed glycosylations, radical catalyzed glycosylations, asymmetric catalytic carbohydrate functionalizations and new glycosylation modalities will be examined.

## Graphical abstract

The recent developments in catalytic stereoselective carbohydrate synthesis are presented. The converging synthetic trajectories of the catalytic and carbohydrate communities are discussed, including impactful case studies exemplifying noncovalent catalysis, radical catalysis, asymmetric catalysis and new catalytic modalities.

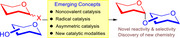

## 1 Introduction

**Figure 1:**
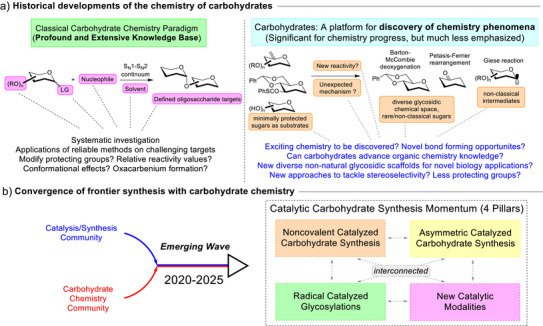
a) Historical development of carbohydrate synthesis: The classical carbohydrate chemistry paradigm and the less emphasized exploitation of carbohydrates as a platform for chemistry discovery. b) Convergence of the catalysis and the carbohydrate community leading to novel conceptual pillars in catalytic carbohydrate synthesis.

The modern science of carbohydrate synthesis is a fascinating and inclusive space where diverse branches of chemistry intersect. Indeed, current knowledge in carbohydrate chemistry had progressed tremendously for over more than a century since the pioneering work from Michael,^[^ ^1^ ^]^ Fischer,^[^ ^2^ ^]^ Königs, and Knorr,^[^ ^3^ ^]^ and later by Helferich in using heavy metals on glycosyl halides.^[^ ^4^ ^]^ Mainstream carbohydrate chemistry^[^ ^5^, ^6^, ^7^, ^8^, ^9^ ^]^ had profoundly flourished in the last 15 years (Figure 1a, left panel), with a strong focus on understanding glycosyl substrate influences. This can be traced back to ground breaking works by Lemieux and co‐workers,^[^ ^10^ ^]^ the watershed development of trichloroacetimidate (TCA) glycosyl donors by Schmidt,^[^ ^11^, ^12^ ^]^ the development of the robust *N*‐phenyltrifluoroacetimidate (PTFAI) donors by Yu,^[^ ^13^ ^]^ the understanding of the arming/disarming nature of protecting groups,^[^ ^14^, ^15^ ^]^ conformational effects,^[^ ^16^, ^17^, ^18^ ^]^ the relative reactivity values of glycosyl donors,^[^ ^19^, ^20^ ^]^ the reactivity of glycosyl acceptors,^[^ ^21^ ^]^ design of novel glycosyl donors with more efficient activation of the leaving group^[^ ^22^, ^23^, ^24^, ^25^ ^]^ and also deepening understanding of the S_N_1‐S_N_2 continuum which best explains the mechanistic manifold of *O*‐glycosylations.^[^ ^26^, ^27^ ^]^ Due to space constraints, many more impactful contributions are unable to be mentioned. These collective efforts had without doubt resulted in predictable and reliable solutions to access oligosaccharides. These strategies had further advanced to the stage of automations either through iterative or one‐pot protocols.^[^ ^28^, ^29^, ^30^, ^31^, ^32^ ^]^

What's less conspicuous in the classical carbohydrate chemistry narrative, but worthy of emphasis, is that the chemistry of carbohydrates is an excellent incubator to advance fundamental synthetic knowledge. This vital facet where frontier organic synthesis intersect with carbohydrates as a unique platform for chemistry discovery possesses deep historical roots (Figure 1a, right panel). For instance, many well‐known named reactions were discovered upon the basis of investigating carbohydrates. This include the Barton‐McCombie deoxygenation,^[^ ^33^ ^]^ the Giese reaction^[^ ^34^ ^]^ and mechanistic studies by Crich^[^ ^35^, ^36^, ^37^ ^]^ which contributed substantially to early days radical chemistry.^[^ ^38^ ^]^ Furthermore, the “Petasis–Ferrier Rearrangement” which left a footprint in total synthesis can be traced back to the Ferrier II rearrangement using monosaccharides.^[^ ^39^, ^40^, ^41^ ^]^ Pivotal concepts such as the (*exo*)anomeric effects had imprinted an indelible mark on the broad topic of stereoelectronic effects.^[^ ^42^, ^43^ ^]^ These historical advances reflect the deeply interwoven ties between carbohydrate synthesis and organic synthesis development.^[^ ^44^, ^45^ ^]^

A renaissance emerged in the last 5 years, where the underexploited interfacial space between carbohydrate chemistry and frontier catalysis had ignited immense attention amongst the broader community of chemists (Figure 1b).^[^ ^46^ ^]^ Motivated by a curiosity‐driven approach,^[^ ^47^ ^]^ this trend had spurred excitement amongst a growing body of catalysis as well as carbohydrate researchers. Many of these cross‐disciplinary endeavors had facilitated the discovery of counter‐intuitive mechanisms, revealed unexpected reactivity and selectivity, minimized the usage of protecting groups in oligosaccharide synthesis and opened up fertile opportunities to tackle fundamental stereoselectivity issues.

Several excellent reviews had discussed specific sub‐components of this newly emerging frontier.^[^ ^48^, ^49^, ^50^, ^51^, ^52^, ^53^, ^54^, ^55^, ^56^, ^57^, ^58^, ^59^, ^60^, ^61^ ^]^ This include those that detailed the exploitation of noncovalent interactions,^[^ ^48^, ^49^, ^50^ ^]^ radical catalysis^[^ ^51^, ^52^, ^53^, ^54^, ^55^, ^62^, ^63^, ^64^, ^65^, ^66^ ^]^ or chiral reagents in carbohydrate synthesis.^[^ ^47^, ^56^, ^58^, ^61^, ^67^ ^]^ Due to the fast‐paced explosion of new developments in the last 3 years, an overarching perspective of this exciting era of catalytic discovery in stereoselective carbohydrate synthesis is now necessary to illuminate this domain for the benefit of the general synthetic readership.

The minireview hence aims to offer a timely consolidation of the latest key catalytic advancements in this new wave of progress. Owing to the sheer volume of reports that had emerged, recent landmark contributions with more focus on examples from the last 3 years will be hand‐picked and critically examined to present an aerial perspective of the interdisciplinary impact of modern catalysis in the carbohydrate chemistry landscape, and vice versa. This minireview will be further partitioned into the following 4 conceptual pillars (Figure 1b): Noncovalent catalyzed glycosylation, radical catalyzed glycosylation, asymmetric catalyzed carbohydrate functionalization and new catalytic modalities in carbohydrate synthesis. An underlying philosophy guiding this exposition is to highlight the strength of the interconnected nature of these concepts. In contrast to prior excellent reviews that approached carbohydrate chemistry from a glycosyl substrate‐based paradigm,^[^ ^5^, ^6^, ^8^, ^9^, ^21^, ^68^, ^69^ ^]^ this review differentiates itself by illuminating innovations using state‐of‐the‐art concepts in catalytic carbohydrate synthesis from the vantage point of a catalysis trained researcher.

## 2 Noncovalent Catalyzed Glycosylation/Carbohydrate Functionalization

**Figure 2:**
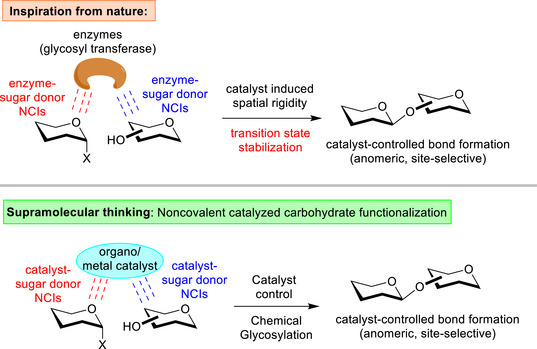
The noncovalent catalyzed strategy in carbohydrate synthesis.

The notion of exploiting weak noncovalent interactions (NCIs)^[^ ^70^, ^71^, ^72^, ^73^, ^74^ ^]^ to steer the stereoselectivity outcome of chemical glycosylations is a relatively new conceptual development (Figure 2).^[^ ^48^, ^49^, ^50^ ^]^ Despite the fact that glycosyl transferases/hydrolases harness NCIs to construct/cleave glycosidic linkages with unparalleled precision,^[^ ^75^ ^]^ striving to realize this analogously in chemical glycosylations had only gained traction recently.^[^ ^48^, ^49^, ^50^, ^76^ ^]^ Major developments of NCI‐based carbohydrate synthesis can be broadly summarized to the harnessing of hydrogen bonding catalysts,^[^ ^71^, ^77^, ^78^, ^79^, ^80^ ^]^ σ‐hole donor catalysts^[^ ^81^, ^82^, ^83^, ^84^, ^85^, ^86^, ^87^, ^88^, ^89^ ^]^ and the tapping of secondary π‐interactions^[^ ^73^ ^]^ to control stereoselectivity in the selectivity determining step.

### 2.1 Hydrogen Bonding Catalysis in Catalytic Glycosylations

**Figure 3:**
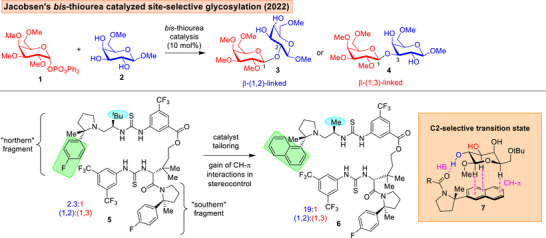
Jacobsen's use of *bis*‐thiourea catalysis to tackle challenging site‐selective glycosylations.^[^ ^98^ ^]^

Pioneering work by the group of Jacobsen in the usage of chiral *bis*‐thiourea hydrogen bonding donors (HBD) as catalysts had found immense utility in chemical glycosylations, although it is essential on the same note to recognize Fairbanks^[^ ^90^ ^]^ and Nargony^[^ ^91^, ^92^, ^93^ ^]^ early contributions in the HB catalyzed domain using phosphoric acids. Since its introduction in 2017 as a glycosylation tool,^[^ ^94^ ^]^ macrocyclic and non‐cyclic variants of HBDs had been employed in a diverse array of glycosylations involving mainly glycosyl phosphate donors.^[^ ^95^, ^96^, ^97^ ^]^ Earlier reported instances disclosed the capability of *bis*‐thiourea catalysts to achieve catalyst controlled β‐1,2‐*cis*‐furanosylations^[^ ^97^ ^]^ and β‐1,2‐*cis*‐mannosylations^[^ ^96^ ^]^ through networks of hydrogen bonds between the thiourea motifs, the glycosyl acceptor and the oxygens on the phosphate leaving group. A prominent feature of a 2022 report involved the concomitant surmounting of anomeric and site‐selectivity challenges when polyol nucleophiles were employed, using a precisely tailored *bis*‐thiourea **5** or **6** (Figure 3).^[^ ^98^ ^]^ The authors discovered that modification of the “northern arylpyrrolidine fragment” to incorporate a napthyl and an alanine residue led to elevation of 2‐OH site‐selectivity, which was attributed to secondary CH‐π interactions.

**Figure 4:**
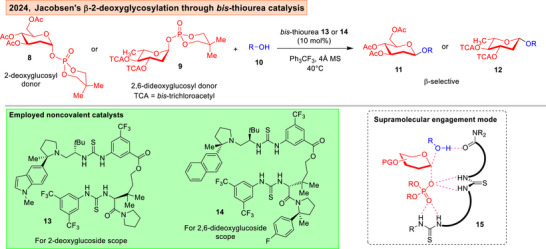
Jacobsen's use of *bis*‐thiourea catalysis to achieve β‐2‐deoxyglucosylations and 2,6‐dideoxyglucosylations.^[^ ^99^ ^]^

Lately, the Jacobsen group reported the challenging synthesis of β‐2‐deoxyglucosides **11** and 2,6‐dideoxyglucosides **12** using *bis*‐thiourea **13** or **14** (Figure 4).^[^ ^99^ ^]^ Besides attributing the catalytic mode of action to a hydrogen bonding network in **15**, the authors also mentioned that molecular sieves and basic alumina was essential to sequester the phosphoric acid by‐products generated in the glycosylation.

**Figure 5:**
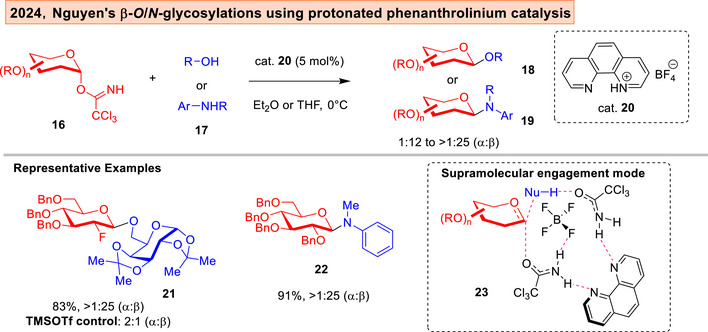
Nguyen's β‐selective glycosylations using cooperative hydrogen bonding interactions.^[^ ^104^ ^]^

Glycosylation advances were also achieved using phenanthroline catalysis by the Nguyen group.^[^ ^100^ ^]^ This catalyst class had been demonstrated previously on pyranosyl^[^ ^101^ ^]^ and furanosyl halides.^[^ ^102^ ^]^ Furthermore, the employment of challenging carboxylic acid acceptors is feasible.^[^ ^103^ ^]^ Significantly, the crucial role of NCIs such as hydrogen bonding was emphasized. A lately published report disclosed a singly protonated phenanthrolinium **20** catalyzing *O,N*‐glycosylations on trichloroacetimidates (Figure 5). The authors postulated a mechanism involving simultaneous interplay of four concurrent hydrogen bonds (HBs) to enforce the β‐stereoselectivity through “cooperative interactions” in **23**.^[^ ^104^ ^]^

**Figure 6:**
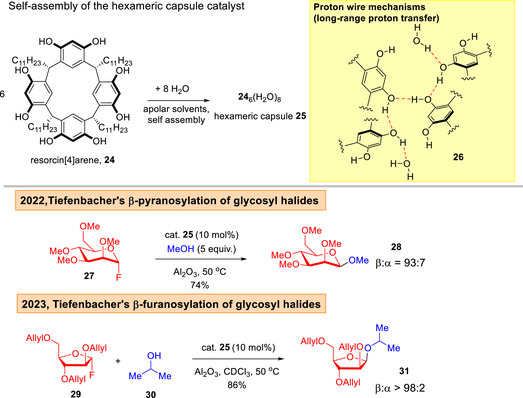
Tiefenbacher's capsule catalyzed glycosylations.^[^ ^105^, ^107^ ^]^

In 2022, the Tiefenbacher group disclosed the use of supramolecular capsule catalysis to achieve unprecedented *O*‐glycosylations starting from glycosyl fluoride donors (Figure 6).^[^ ^105^ ^]^ Grounded in the “proton‐wire” mechanism **26** involving a sophisticated noncovalent long‐range proton transfer found in enzymes,^[^ ^106^ ^]^ β‐selectivity was achieved in both pyranosylations^[^ ^105^ ^]^ and furanosylations^[^ ^107^ ^]^ using resorcin[4]arene **24** derived hexameric capsule **25**. Computations, kinetic and supramolecular experiments led to a postulate of a mechanistic switch from S_N_2 in pyranosyl fluorides to S_N_1 in furanosyl fluorides.^[^ ^108^ ^]^

**Figure 7:**
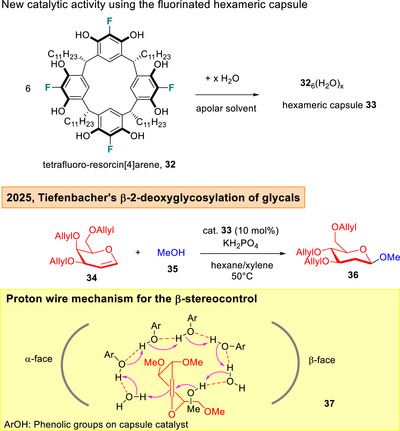
Tiefenbacher's capsule catalyzed β‐2‐deoxyglycosylation.^[^ ^109^ ^]^

Lately, the same research group achieved a breakthrough in the direct β‐2‐deoxyglycosylation of glycals **34** using a tetrafluoro‐resorcin[4]arene capsule **32** (Figure 7).^[^ ^109^ ^]^ This achievement is significant, as many known *O*‐2‐deoxyglycosylation reports showed a strong α‐selectivity preference,^[^ ^110^, ^111^, ^112^, ^113^, ^114^, ^115^, ^116^ ^]^ although it is also pertinent to mention that some β‐selective 2‐deoxyglycosylation protocols had been successfully employed in total synthesis.^[^ ^117^, ^118^ ^]^ Experimental and DFT investigation led to the proposal of a proton‐wire mechanism in **37**,^[^ ^106^ ^]^ which forms the basis of the predominant β‐2‐deoxyglycoside **36** formation.

**Figure 8:**
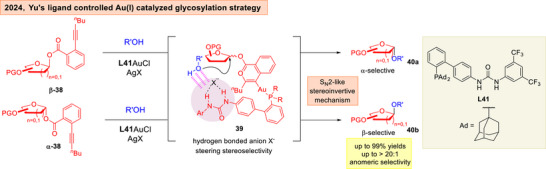
Yu's ligand‐controlled Au(I) catalyzed glycosylation.^[^ ^127^ ^]^ Adapted and modified from Ref. [49] under an open‐access CC‐BY 4.0 License. Copyright 2025, Elsevier Ltd.

The importance of hydrogen bonding control to address carbohydrate selectivity issues is further conceptually broadened to transition metal catalyzed glycosylations.^[^ ^119^, ^120^, ^121^, ^122^, ^123^, ^124^, ^125^, ^126^ ^]^ The Yu group reported in 2025 that by tethering HBD urea motifs onto the JohnPhos ligand, a stereoinvertive Au(I) catalyzed glycosylation using *ortho*‐alkylbenzoate donors **38** can be achieved (Figure 8).^[^ ^127^ ^]^ Through NMR investigations, the authors proposed the triflate or *bis*‐triflimide counteranion could bridge the urea motif with the alcohol functionality on the glycosyl acceptor through HBs in **39**, thus evoking an intramolecular aglycone delivery type process to guide the nucleophile in an anomeric stereoselective fashion.

**Figure 9:**
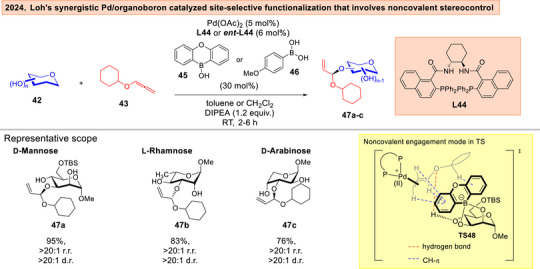
Loh's asymmetric Pd/organoboron catalyzed site‐selective functionalization that involves noncovalent stereocontrol.^[^ ^128^ ^]^ Adapted and modified from Ref. [128] under an open‐access CC‐BY‐NC‐ND 4.0 License. Copyright 2024, Wiley–VCH.

Furthermore, our research group disclosed in 2024 the cruciality of HB alongside with CH‐π interactions in enforcing the multi‐stereoselectivity control in the asymmetric palladium/organoboron catalyzed site‐selective functionalization of carbohydrate polyols **42** with alkoxyallenes **43** (Figure 9).^[^ ^128^ ^]^ It is also vital to mention Taylor's prior seminal works that forms the basis of using organoboron reagents in site‐selective carbohydrate functionalizations.^[^ ^129^, ^130^ ^]^ Through the addition of HB disrupting additives, control experiments on 4‐OH protected substrates, observation of chemical shift perturbations in NMR as well as the use of computations, we brought forth evidence that HB working in synergy with CH‐π in transition state **TS48** are vital in the stereoselectivity determining step.

### 2.2 The σ‐Hole Based Catalytic Strategy in Glycosylations

**Figure 10:**
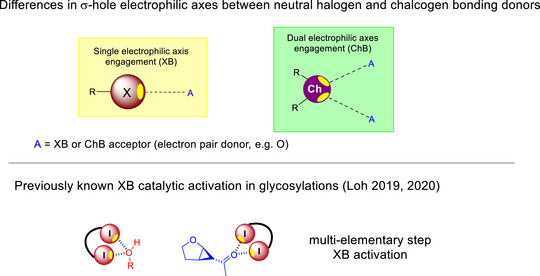
Differences in σ‐holes on neutral XB and ChB donors and XB activation insights from 2019–2020. Adapted and modified from Ref. [50] under an open‐access CC‐BY 4.0 License. Copyright 2025, American Chemical Society, Washington, DC.

There is a surge of attention given to glycosylations empowered by the family of σ‐hole based non‐classical interactions,^[^ ^82^, ^83^ ^]^ such as halogen bonding (XB)^[^ ^131^, ^132^, ^133^ ^]^ and chalcogen bonding (ChB).^[^ ^86^, ^87^, ^89^ ^]^ Underscored by the electronic anisotropy of group VII and VI elements, a highly directional electropositive region on the electrostatic potential surface – known as the “σ‐hole” – offers a geometrically unique non‐protic means of catalytic activation (Figure 10).^[^ ^132^ ^]^ Ever since our pioneering contributions in XB catalyzed strain‐release glycosylations^[^ ^134^ ^]^ and 2‐deoxyglycosylations,^[^ ^116^ ^]^ this σ‐hole based glycosylation strategy is gaining substantial traction in the last 2 years owing to the unexpected robustness of the chalcogen bonding (ChB) catalyzed glycosylation strategy.^[^ ^50^ ^]^

**Figure 11:**
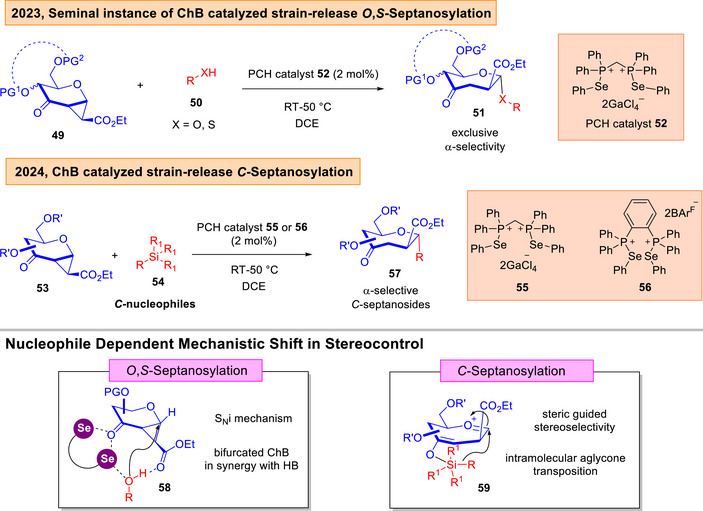
Loh's seminal instances of ChB catalyzed glycosylations to access *O*,*S*,*N*,*C*‐septanosides.^[^ ^142^, ^144^ ^]^ Adapted and modified from Refs. [142, 144] under open‐access CC‐BY 4.0 License and CC‐BY‐NC‐ND 4.0 License. Copyright 2023, American Chemical Society, Washington, DC and Copyright 2024, Wiley–VCH.

Our seminal contribution was showcased in the strain‐release septanosylation to access biologically interesting seven‐ring sugars (Figure 11).^[^ ^135^, ^136^, ^137^, ^138^, ^139^, ^140^, ^141^ ^]^ By the employment of phosphonochalcogenide (PCH) catalysis,^[^ ^89^ ^]^ we discovered that *O*‐/*S*‐septanosides can be accessed with exclusive α‐selectivity through a rare S_N_i (internal nucleophilic substitution) mechanism.^[^ ^142^ ^]^ NMR experiments and DFT analysis led us to the conclusion that an encounter complex **58** involving bifurcated ChB and HB within a noncovalent network is responsible for the excellent stereoselectivity. Counter‐intuitively, by changing the nucleophile choice to a versatile series of silylated nucleophiles,^[^ ^143^ ^]^ we observed that the biologically relevant α,α’‐*C*‐oxepane core can be accessed in a *C*‐septanosylation through an unexpected mechanistic shift.^[^ ^144^ ^]^ Control experiments suggested that the excellent stereoselectivity stems from an intramolecular aglycone transposition pathway through a pentavalent silicon intermediate **59**.^[^ ^145^, ^146^ ^]^

**Figure 12:**
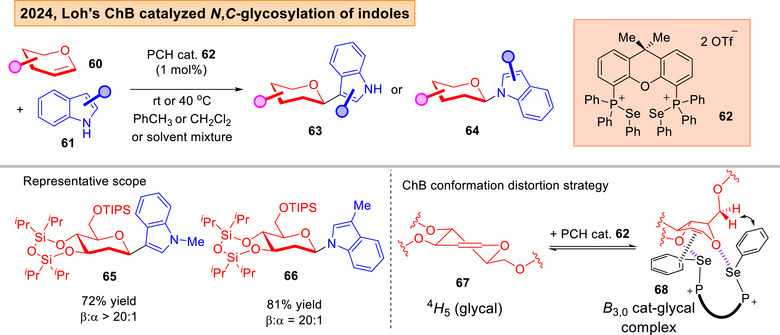
Loh's ChB catalyzed *N*,*C*‐glycosylation of indoles.^[^ ^148^ ^]^ Adapted and modified from Ref. [148] under an open‐access CC‐BY‐NC‐ND 4.0 License. Copyright 2024, Wiley–VCH.

**Figure 13:**
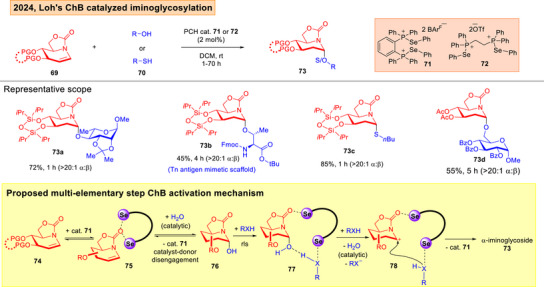
Loh's ChB catalyzed iminoglycosylation that involves multi‐elementary step ChB activation.^[^ ^149^ ^]^ Adapted and modified from Ref. [149] under an open‐access CC‐BY 4.0 License. Copyright 2024, American Chemical Society, Washington, DC.

As part of our programme to access indolyl‐glycoside scaffolds that are prevalent in disease relevant molecules,^[^ ^147^ ^]^ our group discovered that β‐*C*,*N*‐indolyl glycosides **63**–**64** can be stereoselectively synthesized using a glycal conformational distortion strategy (Figure 12).^[^ ^148^ ^]^ Through NMR and DFT studies, we proposed a working hypothesis whereby ChB and π‐interactions worked hand‐in‐hand to distort the ^4^ *H*_5_ conformation in **67** to the *B*_3,0_ conformation in **68**, which facilitated the β‐facile attack of indoles. Moreover, we uncovered a first instance of a multi‐elementary step ChB activation manifold during our study in the *O*/*S*‐iminoglycosylation of iminoglycals **69** (Figure 13).^[^ ^149^ ^]^ Besides evidencing a reversible σ‐hole engagement/disengagement pathway that is distinctive from known 2‐deoxyglycosylations (Figure 13, bottom panel),^[^ ^112^, ^113^ ^]^ we demonstrated that ChB catalysis offered a mild and water tolerant glycosylation strategy that differed from moisture sensitive thiourea catalyzed protocols.^[^ ^111^, ^112^, ^113^ ^]^

**Figure 14:**
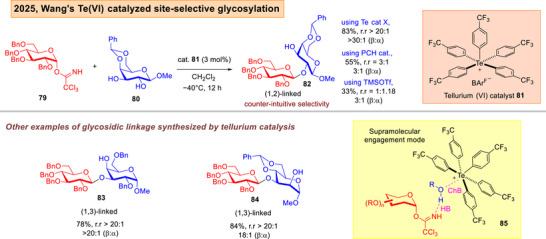
Wang's ChB catalyzed site‐selective glycosylation with TCA donors using tellurium (VI) catalysis.^[^ ^150^ ^]^

**Figure 15:**
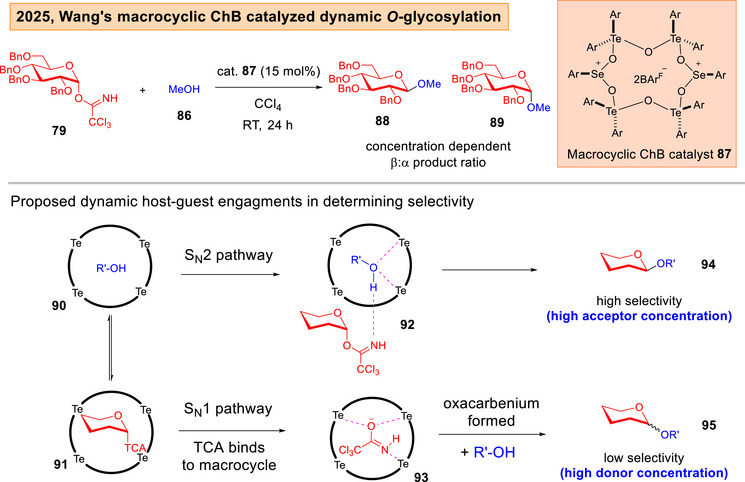
Wang's macrocyclic ChB catalyzed dynamic glycosylation strategy.^[^ ^151^ ^]^

Lately, Wang described the use of ChB based tellurium(VI) catalyst **81** to achieve 2‐OH site‐selective *O*‐glycosylations of diols with trichloroacetimidate (TCA) donor **79** (Figure 14).^[^ ^150^ ^]^ Further, the same group also reported a dynamic glycosylation approach using newly designed macrocyclic chalcogen bonding catalyst **87** on TCA donors, which showed anomeric selectivity elevation, and a shift to an S_N_2 pathway upon increasing the glycosyl acceptor's concentration (Figure 15).^[^ ^151^ ^]^

### 2.3 Critical Evaluation of Noncovalent Catalysis Advances

In a collective spirit to pool in wisdom from the synthetic and carbohydrate communities, it is beneficial to raise awareness of intrinsic substrate and catalyst limitations in these instances. Significantly, we highlight that substrate control still constitutes one of the most challenging aspects to contend with even with modern catalytic advances. In the macrocyclic thiourea catalyzed site‐selective glycosylation (Figure 3),^[^ ^98^ ^]^ the glycosyl donor protection scheme is restricted to less commonly employed methyl or isopropylidene protection. Despite promising site‐selectivity control on the 2‐OH position, the predictability on site‐switchable functionalizations on all possible unprotected alcohols (e.g., 3‐OH, 4‐OH) is still not yet addressed for the broad palette of different saccharides. Further, the macrocyclic thiourea catalyzed β‐2‐deoxyglycosylation for 2,6‐dideoxy‐glycosyl donors required specialized TCA protection,^[^ ^99^ ^]^ and anomeric selectivity in these cases were generally diminished. The glycosyl phosphate donors were also limited to 2‐deoxyglucosyl and 2,6‐deoxyglucosyl congeners. Phenolic acceptors were also providing diminished anomeric selectivity. Improvement of the ease of catalyst preparation and enhancing general availability of the *bis*‐thiourea catalysts such as **13** and **14** are also important factors of consideration by future researchers. Apart from their current non‐commercial availability, synthesis of each of these catalysts involved multi‐step synthetic routes, and the attainment of a reasonable catalyst library needs considerable synthetic effort. Hence, improvement of accessibility either through commercialization or new design of catalysts that require lesser synthetic steps will greatly improve the widespread glycosylation utility of this mode of catalysis.

In Nguyen's protonated phenanthrolinium catalyzed strategy,^[^ ^104^ ^]^ it is noticeable that the glycosyl trichloroacetimidates are limited to perbenzylated protecting group schemes. A key advantage of this mode of catalysis is the facile one‐step synthesis of phenanthrolinium catalysts which can facilitate widespread usage. In the capsule catalyzed method, Tiefenbacher and co‐workers noted pivotal influences in C5 substitution for β‐2‐deoxyglycosylation.^[^ ^109^ ^]^ Hence, using l‐fucal and l‐arabinal substrates led to substantially reduced anomeric selectivity. Additionally, less commonly used allyl protecting groups were generally required. This limitation is due to the size of the confined cavity, which would limit the permissible size threshold of the glycosyl donors. Future efforts could also involve establishing a link between cavity size and the tolerable useful protecting group schemes on the glycosyl donor. The strategy also appears to be sensitive to the diastereomeric identity of the glycosyl donor, as the C4‐epimer *O*‐allyl‐glucal showed no conversion in the absence of acid‐cocatalysts. The nucleophile scope was also generally limited to simple alcohols, and a robust range of more biologically relevant saccharide‐based acceptors on variable nucleophilic OH sites was not demonstrated. This could also be attributed to the restrictions of the confined cavity on the capsule catalyst. On the practical aspects, meticulous synthetic care is further needed prepare the capsule catalysts. This includes careful homogenizing of the catalyst‐solvent mixture, and ensuring anhydrous conditions through molecular sieves addition.

In our ChB catalyzed methods, our research group also encountered protecting group challenges, such as the requirement of more “super‐arming” protection on the cyclopropanated and glycal donors,^[^ ^142^, ^144^ ^]^ although in the iminoglycal cases, disarming groups such as acetyls could be tolerated.^[^ ^149^ ^]^ The phosphonochalcogenides are generally easily attainable in one or two synthetic steps from commercially available phosphines. In Wang's tellurium and macrocyclic ChB catalyzed instances,^[^ ^150^, ^151^ ^]^ the methods were also limited solely to benzyl protection on the glycosyl donor. Further, the achievement of 2‐OH functionalization was only successful in one specified galactosyl diol nucleophile, but not broadly general over a wider range of common sugars. Furthermore, all of the cases involved the study of monosaccharide donors, di‐ and higher level saccharides were not demonstrated. The accommodation of polysaccharide glycosyl donors in future studies will greatly enhance the long‐term usage of these emerging strategies.

All in all, a yet‐to‐be surmounted central challenge will be to identify a versatile but easily synthesizable ideal catalyst that can accommodate all glycosyl donor and acceptors. Acknowledging the non‐triviality of this endeavor, the eventual realization of this will truly achieve unparalleled noncovalent catalyst control in stereoselective carbohydrate synthesis.

## 3 Radical Catalyzed Glycosylations

**Figure 16:**
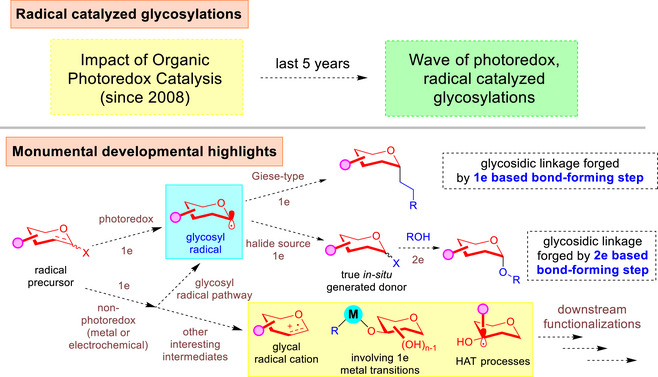
Overview of rapid developments of radical catalyzed carbohydrate synthesis.

In light of the tremendous impact of photoredox catalysis in organic synthesis in the last two decades,^[^ ^152^, ^153^, ^154^, ^155^, ^156^, ^157^, ^158^, ^159^ ^]^ this monumental development had recently found its way into carbohydrate chemistry (Figure 16). A plethora of reviews on radical involved glycosylations are already available,^[^ ^51^, ^52^, ^53^, ^54^, ^55^ ^]^ therefore, the focused purpose of this section is to highlight latest selected advances. Recent literature also revealed that radical involved carbohydrate synthesis can also arise from electrochemical means,^[^ ^160^, ^161^, ^162^, ^163^, ^164^ ^]^ from one‐electron redox transitions in metal catalysis, as well as using photoredox catalysis for hydrogen atom transfer (HAT) processes.^[^ ^165^, ^166^, ^167^, ^168^, ^169^, ^170^, ^171^, ^172^, ^173^, ^174^, ^175^, ^176^ ^]^

### 3.1 Combining Radical Catalysis Concepts with the Noncovalent Strategy in Glycosylations

**Figure 17:**
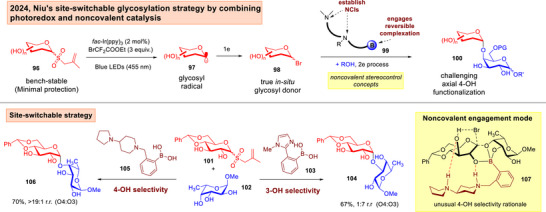
Niu's merger of photoredox and noncovalent organoboron catalysis to synthesize native oligosaccharides.^[^ ^177^ ^]^ Adapted and modified from Ref. [49] under an open‐access CC‐BY 4.0 License. Copyright 2025, Elsevier Ltd.

One of the most defining instances in recent literature is the photoredox catalyzed synthesis of native glycosidic linkages using minimally protected glycosyl donors and acceptors by Niu in 2024 (Figure 17).^[^ ^177^ ^]^ The authors elegantly demonstrated that by using an iridium photocatalyst with a designed aminoboronic acid catalyst **99**, radical activation of the glycosyl allyl sulfone could be realized to access an in‐situ glycosyl halide **98**. Reversible complexation of the aminoboronic acid **105** with the polyol acceptor creates the opportunity for a network of hydrogen bonding interactions in **107** to forge both anomeric and site‐selectivity control.^[^ ^178^ ^]^

**Figure 18:**
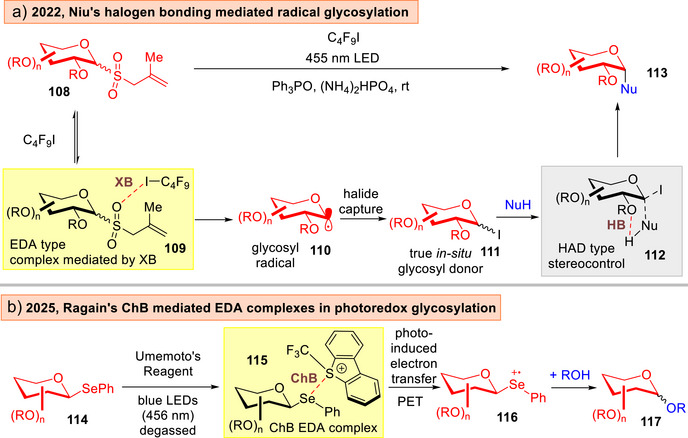
a) Niu's harnessing of XB mediated EDA complexes and HAD in *O*‐1,2‐*cis*‐glycosylations.^[^ ^179^ ^]^ b) Ragain's exploitation of ChB mediated EDA complexes in *O*‐glycosylations.^[^ ^184^ ^]^

The same group showcased in 2022 the merger of XB with photoredox activation using glycosyl allyl sulfones **108** to afford 1,2‐*cis* glycosidic linkages in **113** (Figure 18a).^[^ ^179^ ^]^ The α‐1,2‐*cis*‐stereocontrol was attributed to a hydrogen bonding assisted aglycone delivery (HAD)^[^ ^180^, ^181^, ^182^, ^183^ ^]^ type process in **112**. Recently, the Ragains group capitalized on the dual strategy of photoredox catalyzed and chalcogen bonding activation to form activated EDA complexes **115** of selenoglycosides and the Umemoto's reagent to effect *O*‐glycosylations (Figure 18b).^[^ ^184^ ^]^

**Figure 19:**
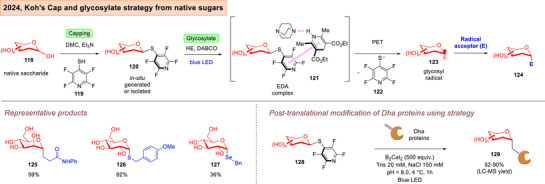
Koh's “*cap and glycosylate*” strategy which harnesses native sugars for *C*,*S*,*Se*‐glycosylation.^[^ ^185^ ^]^

Koh and Davis lately reported an elegant photoredox catalyzed “*cap and glycosylate”* strategy that involved a “traceless activation” (Figure 19).^[^ ^185^ ^]^ This converted an unprotected saccharide **118** to a 2,3,5,6‐tetrafluoropyridine‐4‐thioglycoside **120** in situ. Subsequently, in the presence of a Hantzsch ester (HE), an EDA complex **121** stabilized by π‐interactions, is formed. This culminates in the formation of a glycosyl radical **123**, and subsequent radical attack with a range of coupling partners to access α‐*C*,*Se*,*S*‐glycosides **124**. Significantly, this protocol was amenable to post‐translational modifications on tagged Dha proteins.^[^ ^186^, ^187^ ^]^ It is also noteworthy that the Koh group had previously made important contributions through a iron catalyzed or synergistic iron and nickel catalyzed radical platform to stereoselectively access aryl‐*C*‐glycosides through the crucial formation of a glycosyl radical.^[^ ^188^ ^]^

### 3.2 Advances in Site‐Selective and Site/Reactivity‐Divergent Radical *C*‐Glycosylation Strategies

**Figure 20:**
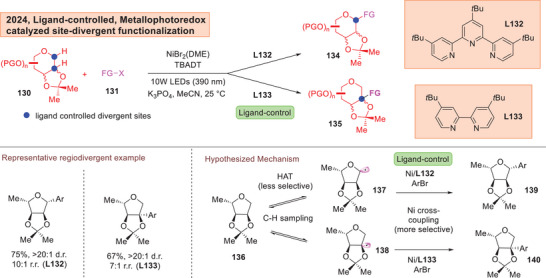
Qi and Kong demonstration of ligand‐divergent C─H functionalizations on 1‐deoxy‐glycosides.^[^ ^189^ ^]^

Qi and Kong lately exploited a metallophotoredox strategy to achieve switchable selectivity on the C1 or C2 position of 1‐deoxyglycosides by judiciously switching between a tripyridyl **L132** or a dipiyridyl **L133** ligand to access C1‐arylated **134** and C2‐arylated **135** respectively (Figure 20).^[^ ^189^ ^]^ Through experiments and computations, the authors attributed the observed regioselectivity to a tight‐knitted combination of bond dissociation energies, polar effects^[^ ^190^, ^191^ ^]^ and steric effects.

**Figure 21:**
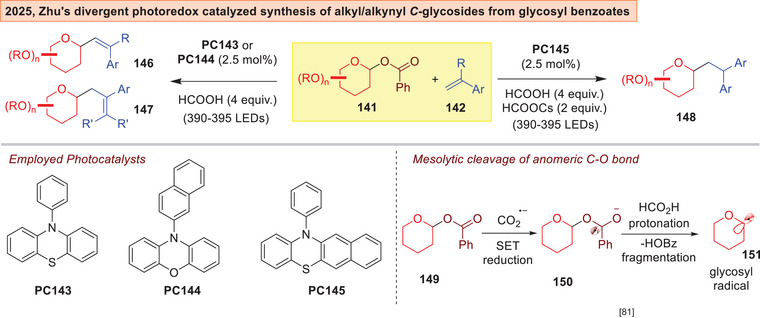
Zhu's photocatalyzed divergent synthesis of alkenyl/alkyl *C*‐glycosides.^[^ ^192^ ^]^

Zhu and co‐workers lately established lately a reductive photoredox divergent and switchable deoxygenative strategy towards alkyl or alkenyl *C*‐glycosides **146**–**148** from easily available glycosyl benzoates **141** (Figure 21).^[^ ^192^ ^]^ This strategy harnesses for the first time CO_2_ radical anions^[^ ^193^, ^194^, ^195^ ^]^ as strong reductants for mesolytic cleavage of the anomeric C─O bond through a protonation/fragmentation process from **150** to access the glycosyl radical **151**.

**Figure 22:**
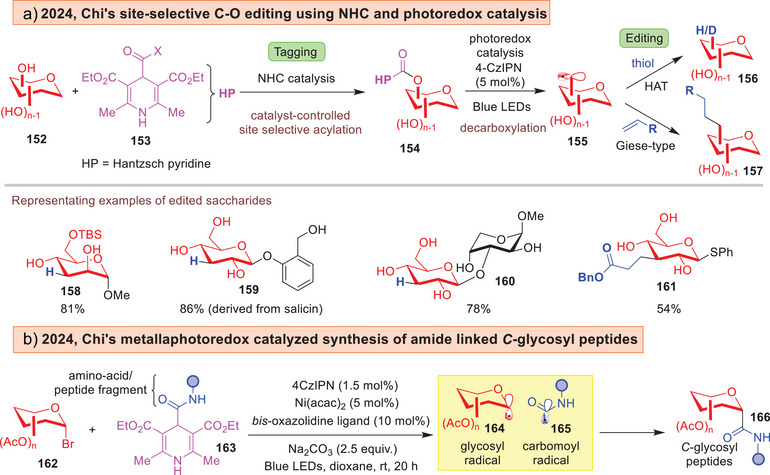
a) Chi's “tagging/editing strategy” by combining NHC and photocatalysis.^[^ ^196^ ^]^ b) Chi's metallaphotoredox catalyzed synthesis of *C*‐glycosyl peptides.^[^ ^197^ ^]^

An instance of saccharide editing was introduced by Chi and co‐workers using a “tagging‐editing” strategy to effect deoxygenation site‐selectively either on the C6 or C3 position (Figure 22a).^[^ ^196^ ^]^ This strategy commences with a selective *N*‐heterocyclic carbene (NHC) catalyzed installation of a dihydropyridyl (DHP) ester to introduce the tag on a defined site in **154**. The subsequent editing step harnesses photoredox catalysis to generate a *C*‐centered radical **155** through decarboxylative β‐scission. Finally, a hydrogen atom transfer or Giese‐type reaction will furnish the deoxygenated/alkylated glycosides **156**–**157**. The Chi group further synthesized amide‐linked *C*‐glycosyl peptides through generating carbomoyl radicals **165** starting from amino‐acid derived dihydropyridine **163** using a 4CzIPN/Ni metallaphotoredox catalytic system (Figure 22b).^[^ ^197^ ^]^

**Figure 23:**
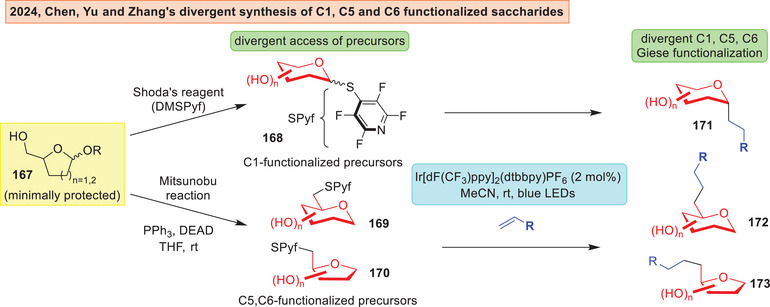
Chen, Yu, and Zhang use of SPyf functionalization for downstream regiodivergent functionalizations.^[^ ^198^ ^]^

Another site‐divergent strategy was introduced by Chen, Yu and Zhang,^[^ ^198^ ^]^ where the authors reported the use of 4‐tetrafluoropyridinylthio (SPyf) moiety as an adaptive activating group that could be attached on C1, C5 and C6 positions of a saccharide **167** (Figure 23). By using the Shoda's reagent^[^ ^199^ ^]^ or employing Mitsunobu conditions, the minimally protected C1, C5, and C6 precursors **168**–**170** can be accessed orthogonally. These radical precursors could then be functionalized photocatalytically to access a divergent array of saccharides **171**–**173**.

### 3.3 Advances in Radical Catalyzed *N*‐Glycosylation Strategies

**Figure 24:**
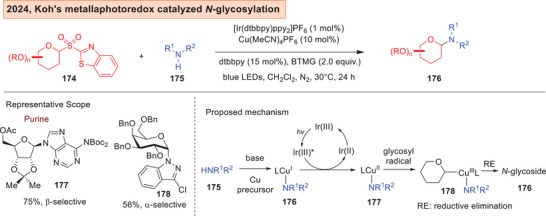
Koh's metallaphotoredox catalyzed radical *N*‐glycosylation.^[^ ^201^ ^]^

There is also an emerging front whereby the generation of glycosyl radicals is harnessed as a synthetic means to access *N*‐glycosides.^[^ ^200^ ^]^ Koh and Chen successfully employed aryl glycosyl sulfones **174** in conjunction with copper catalyzed photoredox catalysis (Figure 24)^[^ ^201^ ^]^ to access wide array of *N*‐glycosides and nucleosides **176** bearing both furanosyl and pyranosyl cores. Mechanistically, the photoredox cycle facilitates the oxidation of the amide‐coordinated Cu^I^ **176** to Cu^II^ **177**. A subsequent radical association with the glycosyl radical yields the Cu^III^ intermediate **178** and a reductive elimination afforded the *N*‐glycosides **176**.

### 3.4 Emerging Radical Catalyzed Glycosylation Strategies that do not Involve Photoredox Chemistry

**Figure 25:**
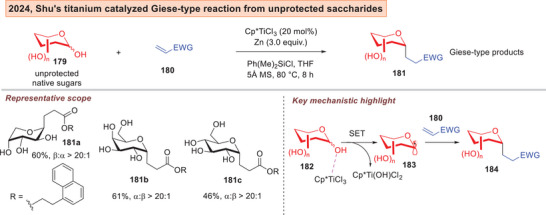
Shu's titanium catalyzed Giese *C*‐glycosylation from unprotected native saccharides.^[^ ^202^ ^]^

Furthermore, non‐photoredox generation of glycosyl radicals by using single electron transitions on metal catalysis are also on the rise. In 2024, the Shu group disclosed a titanium catalyzed Giese type *C*‐glycosylation from readily available unprotected reducing sugars (Figure 25).^[^ ^202^ ^]^ Impressively, no prefunctionalization of the sugar donor **179** was necessary.

**Figure 26:**
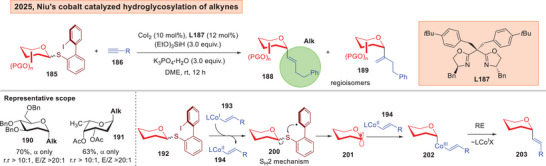
Niu's cobalt catalyzed hydroglycosylation of alkynes.^[^ ^205^ ^]^

Lately, Wang, and Niu reported the use of *ortho*‐iodobiphenyl donors **185** that uses a S_H_2 (bimolecular homolytic substitution)^[^ ^203^, ^204^ ^]^ activation mechanism to afford α‐vinyl‐*C*‐glycosides by means of radical cobalt catalysis (Figure 26).^[^ ^205^ ^]^ Unactivated terminal alkynes **186** can be employed as the *C*‐acceptor, which is then converted to the *E*‐configured alkene **188**. The hydroglycosylation involves the *in*‐situ generation of a Co^I^‐H **193**, which then underwent SET to access a Co^II^ species **194**. A radical rebound achieved the Co^III^ intermediate **202** which culminates in a final reductive elimination to generate the vinyl *C*‐glycosides **203**. The chirality of the ligand was found to be inconsequential to the stereoselectivity preference.

### 3.5 Critical Evaluation of Radical Catalyzed Glycosylation

In spite of fascinating advances in radical catalyzed approaches, it is also pertinent to appreciate considerable challenges in catalyst versus substrate control that still persist in this domain. For instance, the elimination of protecting groups is not homogenously applied across the above‐mentioned methods. In Niu's elegant photocatalyzed approach using minimally protected donors and acceptors,^[^ ^177^ ^]^ strategic protection of certain positions (e.g., 6‐OH on the allyl glycosyl sulfone) is required for the method to be successful. In Niu's^[^ ^179^ ^]^ and Ragains's^[^ ^184^ ^]^ approaches that use XB or ChB in *O*‐glycosylations, perbenzylated protection is still necessary and anomeric selectivity was still modest in the latter.

In *C*‐glycosylation instances by Koh,^[^ ^185^ ^]^ as well as in other above‐described cases by Chi,^[^ ^197^ ^]^ Chen, Yu, and Zhang,^[^ ^198^ ^]^ Zhu,^[^ ^192^ ^]^ Niu^[^ ^205^ ^]^ as well as Shu,^[^ ^202^ ^]^ the general stereochemical approach still hinges upon the Giese pioneering formation of a glycosyl radical through different catalytic strategies, followed by it's stereoelectronic controlled interception with an activated/non‐activated alkene/alkyne via an α‐facial trajectory. In specified cases, such as the report by Shu,^[^ ^202^ ^]^ native saccharides could be employed, but glycosyl donors in varying extensiveness of protection remained dominant in other reports. Thus, it is also essential that the commonly articulated mild, and “protecting group free” benefits of photoredox catalyzed glycosylations be objectively viewed with these nuances in place. New methods that can provide consistency in protecting group schemes will also be in high demand. Recognizing the availability of the β‐selective “anti‐Giese” approach in limited reports,^[^ ^206^ ^]^ β‐selective radical catalyzed glycosylations still remains the much lesser explored domain. In Koh's radical *N*‐glycosylation strategy,^[^ ^201^ ^]^ anomeric selectivity is still highly nucleophile and glycosyl donor dependent. For instance, adenosine and sulfonamide based nucleophiles alongside with d‐xylose derived donors gave diminished anomeric selectivity.

On the site‐selectivity front, notwithstanding Qi and Kong's excellent contribution to C1/C2 site‐divergency,^[^ ^189^ ^]^ there is a strong element of substrate design as a 2,3‐isopropylidene protection is dominant across the substrate scope. Additionally, the donor scope is also limited to a confined palette of monosaccharides but not on polysaccharides. Further progress in using polysaccharides for site‐divergent functionalizations of the carbon skeletal framework of sugars will substantially advance this area. Careful inspection also revealed that nickel catalyst loadings need to be substantially elevated to 20 mol% for C1‐functionalizations as compared to 10 mol% for C2‐functionalizations. This suggests that consistency in catalytic conditions is still a challenging aspect that is yet to be fully overcome.

Niu's synergistic photoredox/amino‐organoboron catalyzed strategy^[^ ^177^ ^]^ has definitely opened up new avenues for 2‐OH/3‐OH switchability, but close scrutiny also revealed at the challenging 2‐OH axial selectivity of mannoside acceptors is comparatively modest (1:4–1:6 C3:C2) to the 3‐OH selectivity. However, the use of the non‐commercially available aminoboronic acid **105** for the prized 2‐OH switchable functionalization may hinder widespread use due to catalyst accessibility considerations in conjunction with relatively lower 2‐OH site‐selectivity. Notwithstanding, a clear advantage regarding the use of photoredox radical catalyzed strategies is the commercial availability of photocatalysts from many chemical suppliers, and the convenient procurement of variable wavelength LEDs for photoredox laboratory set‐ups.

## 4 Asymmetric Catalysis in Glycosylation and Glycofunctionalization

**Figure 27:**
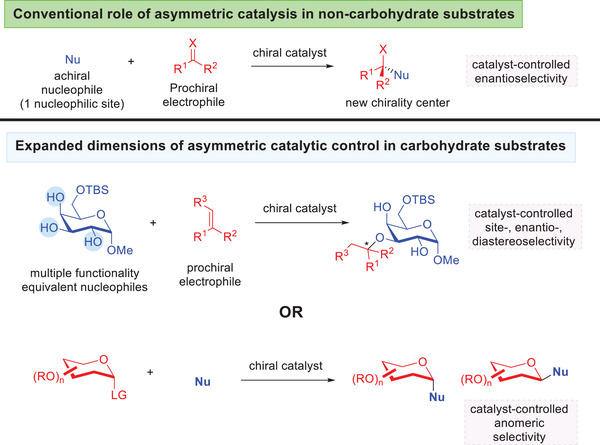
Broadened role of asymmetric catalytic control in stereoselective carbohydrate synthesis.

Asymmetric catalysis is conventionally associated with chirality creation from prochiral substrates by employing a sub‐stoichiometric amount of a chiral catalyst (Figure 27).^[^ ^207^ ^]^ This framework is now experiencing a broadening of definition in the realm of carbohydrate synthesis,^[^ ^61^ ^]^ where the chirality information of the catalyst could instead be tapped upon to address challenges such as regioselective functionalization,^[^ ^56^, ^58^ ^]^ or to simultaneously forge bonds diastereoselectively on the anomeric carbon.^[^ ^48^ ^]^ It is also important to credit early prominent contributions by Miller,^[^ ^208^ ^]^ Dong,^[^ ^209^ ^]^ Niu^[^ ^210^ ^]^ and Tang.^[^ ^211^ ^]^

### 4.1 Employing Chiral Ligands for Site‐ and Anomeric Selectivity Control

**Figure 28:**
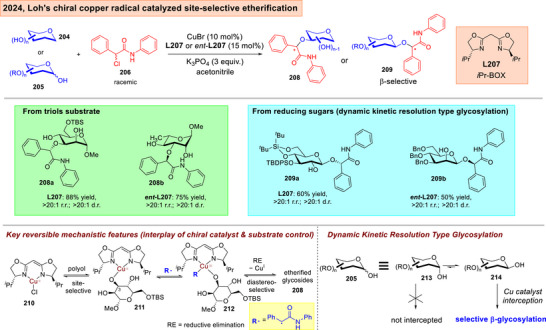
Loh's asymmetric copper radical catalyzed enantioconvergent site‐selective etherifications and dynamic kinetic resolution of carbohydrate polyols.^[^ ^212^ ^]^ Adapted and modified from Ref. [212] under an open‐access CC‐BY‐NC‐ND 4.0 License. Copyright 2024, Wiley–VCH.

Our group lately published an enantioconvergent copper radical catalyzed site‐selective etherfication of minimally protected saccharides (Figure 28).^[^ ^212^ ^]^ This strategy taps upon emerging chiral copper radical catalysis in C─O bond formations,^[^ ^213^, ^214^, ^215^ ^]^ and mechanistically commences with the enantioconvergent formation of a prochiral *C*‐centered radical from α‐haloamides.^[^ ^216^ ^]^ Through subsequent reversible elementary steps that involve radical rebound onto a Cu^II^ species **211** to form a high valent Cu^III^ intermediate **212**, a final reductive elimination installs the carboxamide with precise stereocontrol on site‐ and diastereoselectivity. Significantly, this method does not require co‐catalysis by an organoboron reagent.^[^ ^217^, ^218^, ^219^, ^220^ ^]^ Moreover, we showed that a dynamic kinetic resolution type glycosylation (see Section 5) can be achieved using reducing sugars **205** to yield **209a‐b**.

**Figure 29:**
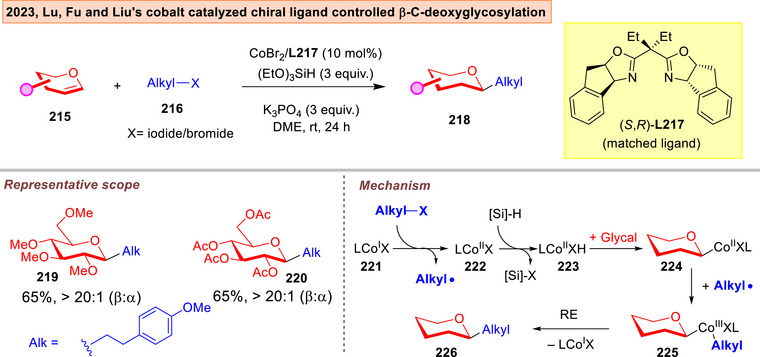
Cobalt catalyzed, chiral ligand‐controlled *C*‐2‐deoxyglycosylations by Lu, Fu and Liu.^[^ ^221^ ^]^

In 2023, Lu, Fu, and Liu reported that the more challenging β‐*C*‐glycosides can be synthesized directly from glycals and alkyl halides via chiral cobalt radical catalysis (Figure 29).^[^ ^221^ ^]^ By exploiting *bis*‐oxazolidine ligand **L217**,^[^ ^222^, ^223^, ^224^ ^]^ a broad series of glycals **215** can be employed with generally excellent β‐selectivity. The use of the opposite enantiomer of the ligand resulted in vastly diminished yields and anomeric selectivity, indicating a matched/mismatching effect. The mechanism involves the hydrometallation of a Co^II^ hydride **223**, which then recombines with an alkyl radical to form a β‐C‐Co^III^ bond at the anomeric center in **225**. This eventually underwent reductive elimination to generate the β‐*C*‐2‐deoxy‐glycosides **226**.

**Figure 30:**
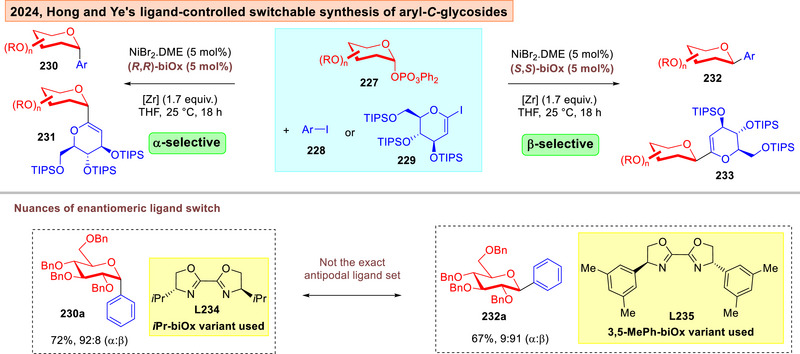
Zirconoaziridine mediated asymmetric nickel catalyzed anomeric switchable *C*‐glycosylation.^[^ ^225^ ^]^

Hong and Ye disclosed in 2024 a zirconoaziridine‐mediated asymmetric nickel catalysis in the ligand‐controlled switchable synthesis of aryl‐*C*‐glycosides **230**–**233** (Figure 30).^[^ ^225^ ^]^ Using glycosyl phosphates **227** and iodides **228**–**229** as coupling partners, the authors demonstrated that the judicious use of enantiomeric variants of chiral *bis*‐oxazoline (biOx) ligands **L234‐L235** can led to modulable α‐ and β‐selectivity outcomes.

**Figure 31:**
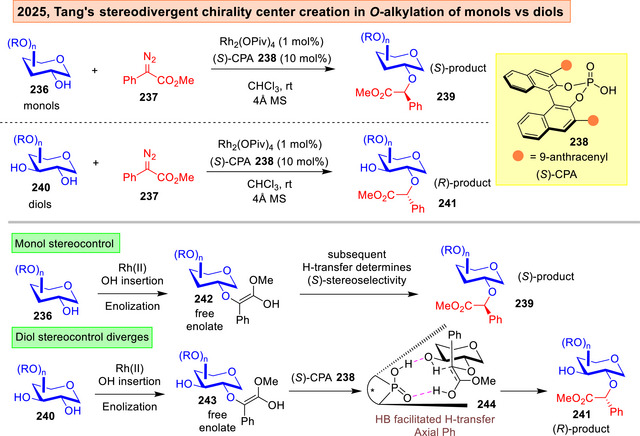
Tang's stereodivergent Rh(II) and chiral phosphoric acid catalyzed *O*‐alkylation.^[^ ^226^ ^]^

Tang lately reported chirality center generation on functionalized sugars using a Rh^(II)^ carbenoid insertion platform in conjunction with a chiral phosphoric acid (CPA) **238**.^[^ ^226^ ^]^ The authors discovered an interesting mechanistic divergence: Mono‐alcohol substrates **236** generated the *S*‐configured stereocenter, while diols **240** gave the *R*‐configured stereocenter (Figure 31). In the mono‐alcohol instance, a CPA controlled proton transfer to the free enolate intermediate was proposed. In the diol instance, the CPA would enforce an axial conformation on the phenyl group that facilitated the establishment of a HB‐bridge in **244**. This culminates in a catalyst‐controlled proton delivery onto the prochiral carbon to access the *R*‐configured diastereomer **241** instead.

**Figure 32:**
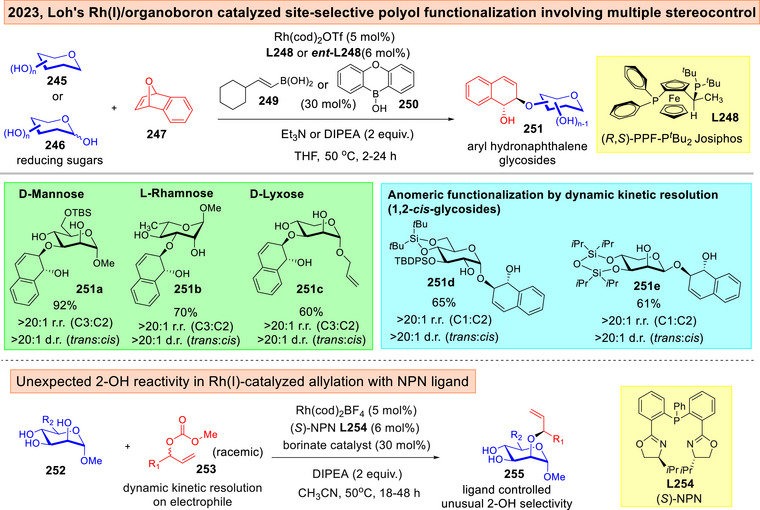
Loh's Rh(I)/organoboron site‐selective polyol functionalization involving multiple stereocontrolling elements.^[^ ^227^ ^]^ Adapted and modified from Ref. [227] under an open‐access CC‐BY 4.0 License. Copyright 2023, Springer Nature.

Our research group showcased in 2023 a synergistic chiral Rh^(I)^ catalysis and organoboron catalyzed strategy that involved a rare blend of multi‐stereocontrol to access aryl‐hydronapthalene glycoside scaffolds **251** (Figure 32).^[^ ^227^ ^]^ Minimally protected carbohydrate diols or triols **245**–**246** were employed as nucleophiles and *meso*‐oxanorbornadienes **247** as electrophiles. A crucial facet of this method lies in its precision to tackle multiple enantio‐, diastereo‐ and site‐selectivity concomitantly within a single bond forming step. Significantly, this catalytic system is also amenable towards dynamic kinetic resolution control on reducing sugars to access the challenging 1,2‐*cis*‐glycosides **251d‐e**. By expanding our investigation to allylic carbonates **253**, the employment of NPN ligand **L254** alongside with dynamic kinetic resolution control on the racemic electrophile **253** resulted in a counter‐intuitive allylation at the 2‐OH position instead.

### 4.2 Critical Evaluation of Asymmetric Catalytic Strategies

In view of the above advances, there are definitely formidable substrate‐based issues that had yet to be fully resolved. For instance, our chiral copper radical catalyzed case,^[^ ^212^ ^]^ as well as our Rh(I) catalyzed site‐selective functionalization^[^ ^227^ ^]^ gave predominantly 3‐OH functionalized products. This reflected the 3‐OH intrinsic bias in such protocols, although we emphasize the undeniable strong influences of chiral ligands through mismatching controls in establishing excellent stereoselectivity. Further, we also noted in our control experiments that 6‐OH protection is often critical, as also seen in Niu's recent photoredox instances,^[^ ^177^ ^]^ as well Tang's lately reported Rh(II) carbenoid insertion case using a chiral phosphoric acid co‐catalyst.^[^ ^226^ ^]^ All these instances reflected deep understanding of the subtleties of minimal protection schemes that cannot yet be fully eradicated in catalytic design.

In Hong and Ye's ligand control switchable synthesis of *C*‐aryl‐glycosides,^[^ ^225^ ^]^ the switchability of enantiomeric ligand pairs is however, not as straightforward because the fine‐tuning of the ligand's substituents was required. Further, our observation that NPN ligand was capable of toggling a 2‐OH selectivity^[^ ^227^ ^]^ further suggested that successful site‐switchability could also arise by considering completely different ligand architectures, apart from approaches that switch the enantiomeric identities of the chiral ligand.^[^ ^210^ ^]^

The correct matched/mismatched combination is further critical in both site‐selective or anomeric‐selective instances. In Lu, Fu, and Liu's asymmetric cobalt catalyzed β‐*C*‐deoxyglycosylation, a switch towards the (*R*)‐congener of the ligand gave substantially diminished anomeric selectivity.^[^ ^221^ ^]^ In all of our research group's contributions, we also noted clear influence of the matching ligand/sugar substrate pairing for all dimensions of stereoselectivity.^[^ ^128^, ^212^, ^227^ ^]^ An obvious gap in this arena is the general lack of studies on varied and biologically relevant di‐ or oligosaccharide sequences as polyol substrates, although isolated examples of simple di‐ or tri‐saccharides exist.^[^ ^221^, ^226^ ^]^ Further investigations in more complex oligosaccharides would greatly enhance the robustness of the asymmetric catalytic concepts on carbohydrate functionalizations. As privileged chiral ligands that were previously developed for enantioselective functionalizations are easily purchasable from commercial sources, this ensures good chiral catalyst accessibility for future progression in this area.

## 5 New Catalytic Modalities

### 5.1 Newly Emerging Catalytic Strategies

**Figure 33:**
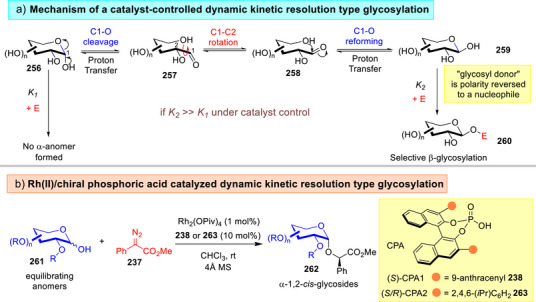
a) Mechanism of a dynamic kinetic resolution type glycosylation, b) Tang's Rh(II)/chiral phosphoric acid catalyzed dynamic kinetic resolution type glycosylation strategy.^[^ ^232^ ^]^

There is a notable emergence of new paradigms for catalytic glycosylations. The first area is the harnessing of catalytic control in the dynamic kinetic resolution^[^ ^228^ ^]^ type glycosylation on reducing sugars (Figure 33a). This unconventional concept presents a mechanistic “Umpolung” from the standard glycosylation, where the glycosyl donor reverses its polarity to function as a nucleophile. The catalyst will selectively intercept one of the two equilibrating anomers **256**, **259** that is rapidly undergoing mutarotation, eventually funnelling the mechanism to a defined anomer. It is also important to credit notable early non‐catalytic works from Schmidt^[^ ^11^ ^]^ and Zhu^[^ ^229^ ^]^ who employed classical thermodynamic or kinetic control, as well as cesium chelation control in such “Umpolung” strategies.

Besides the examples reported by us in Section 4,^[^ ^212^, ^227^ ^]^ and early contributions by Takemoto,^[^ ^230^, ^231^ ^]^ Tang reported in 2023 the employment of Rh^(II)^/CPA catalyzed dynamic kinetic stereoselective glycosylation using the carbenium insertion platform (Figure 33b).^[^ ^232^ ^]^ The authors showcased the amenability of their alkylative strategy over a broad array of reducing sugars ranging from mono‐ to trisaccharides to afford the highly demanded α‐1,2‐*cis*‐glycosides **262** with impressive anomeric selectivities. Intriguingly, the authors emphasized the concurrent importance of noncovalent π–π interactions imparted by the C2‐OBn group to enforce the anomeric selectivity.^[^ ^48^ ^]^

**Figure 34:**
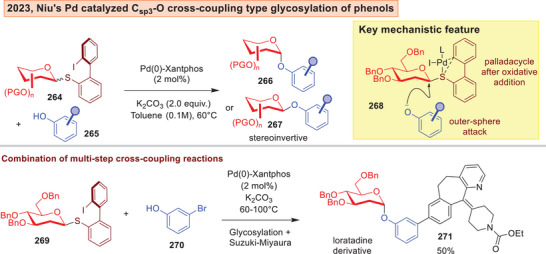
Pd catalyzed C_sp3_‐O cross‐coupling type glycosylation with phenols by Niu.^[^ ^239^ ^]^

Cross‐coupling type reactions^[^ ^233^, ^234^, ^235^, ^236^, ^237^, ^238^ ^]^ is also carving new paths in carbohydrate synthesis. The group of Niu described in 2023 a powerful instance of a C*_sp3_*‐O cross‐coupling type glycosylation with phenols **265** by accessing a Pd(II) intermediate via a donor that contains an aryl‐iodide‐sulfide leaving group **264** (Figure 34).^[^ ^239^ ^]^ Differing from classical aryl‐halide based electrophiles, this strategy harnesses instead an outer sphere attack of the phenoxide nucleophile in **268** to forge the inverted *O*‐glycosidic linkage in **266**–**267**. This strategy can also be combined with the Suzuki‐Miyaura coupling in a multi‐step process to access biologically interesting derivatives such as **271**.

**Figure 35:**
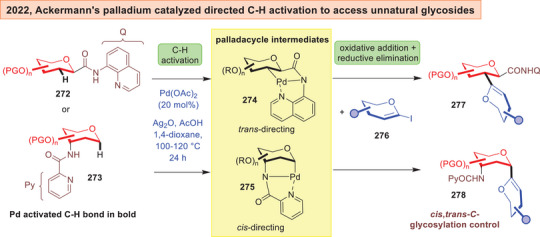
Pd catalyzed directed CH‐activation to access unnatural *C*‐glycosides.^[^ ^240^ ^]^

Ackermann reported in 2022 the synthesis of unnatural *C*‐disaccharides **277**–**278** from quinolinyl/pyridyl tethered saccharides **272**–**273** and iodo‐glycals **276** using palladium catalyzed C*_sp3_*‐H activation (Figure 35).^[^ ^240^ ^]^ The key mechanism involves formation of a heterocycle directed palladacycle **274** or **275** that can engage with oxidative addition with **276**, which finally culminates in the C─C bond formation through a reductive elimination.

**Figure 36:**
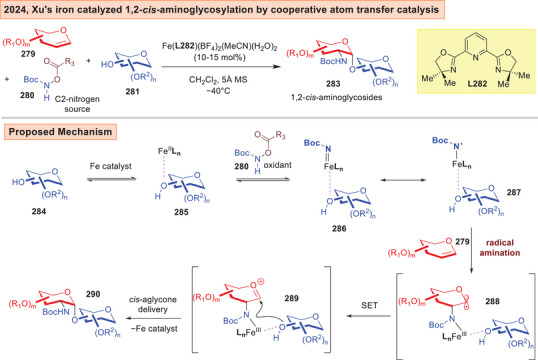
Fe catalyzed 1,2‐*cis*‐aminoglycosylation by cooperative atom transfer catalysis.^[^ ^241^ ^]^

A robust aglycone delivery method using “*cooperative atom transfer catalysis*” to generate biologically important 1,2‐*cis*‐aminoglycosides from glycals was lately reported by Xu (Figure 36).^[^ ^241^ ^]^ This radical involved catalytic strategy leverages the formation of an iron‐saccharide‐aminyl radical species **287** from acyloxycarbamate **280** through an oxidative process. This then undergoes radical addition to form a 2‐amidoglycosyl radical **288**. This species converts to an oxacarbenium ion **289** after a SET, and a subsequent Fe coordinated aglycone delivery process transfers the glycosyl acceptor to establish the α‐glycosyl linkage in **290**.

**Figure 37:**
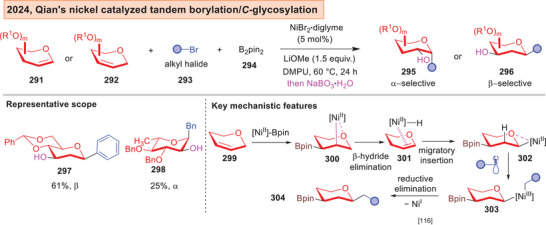
Qian's nickel catalyzed tandem borylation/*C*‐glycosylation.^[^ ^242^ ^]^

Recently, Qian and co‐workers reported a nickel catalyzed tandem carboboration on glycals (Figure 37).^[^ ^242^ ^]^ By employing 1,2‐ or 2,3‐glycals **291–292**, an array of 1,2‐ or 1,3‐difunctionalized sugars **295**–**296** can be accessed. Furthermore, the presence of a C‐B bond offered versatility for downstream derivatizations, such as in C─C, C─H, C─N bond formations. This reaction is proposed to proceed through Ni^I^/Ni^II^/Ni^III^ redox cycling, that involved a series of β‐hydride elimination, migratory insertion, radical association and finally reductive elimination steps to generate the difunctionalized sugar **304**.

**Figure 38:**
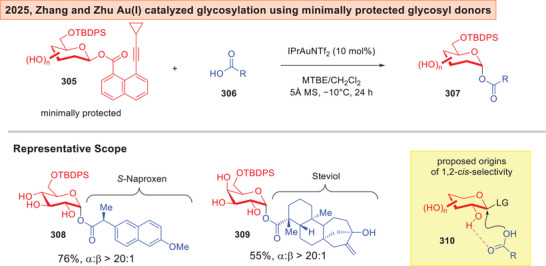
Au(I) catalyzed glycosylation with carboxylic acid that employs minimally protected alkynyl glycosyl donors.^[^ ^243^ ^]^

Last but not least, Zhang and Zhu described lately a mild Au(I) catalyzed glycosylation^[^ ^22^ ^]^ of minimally protected glycosyl‐8‐alkynyl‐1‐napthoate **305** with carboxylic acids **306** to synthesize 1,2‐*cis*‐glycosides (Figure 38).^[^ ^243^ ^]^ The method can further be utilized to access biologically interesting derivatives **308**–**309** containing *S*‐Naproxen and Steviol respectively. This report thus constitutes a notable advance where 2‐electron based Au(I) catalysis is compatible with minimally protected glycosyl donors. The 1,2‐*cis*‐selectivity is proposed to originate from a HB between the 2‐OH group and the carboxylic acid based on NMR evidence as seen in **310**.

### 5.2 Critical Evaluation of New Catalytic Modalities

Similarly to previous sections, substrate‐based caveats exist in these new catalytic modalities that have to be appreciated. First, for the dynamic kinetic resolution type glycosylations, fully protected reducing sugars had to be to employed.^[^ ^232^ ^]^ Further, the 2‐deoxy‐donors led to diminished diastereoselectivity during the chirality creation, suggesting a key role of the 2‐OH protecting group. For Niu's C*_sp3_*‐O cross‐coupling type glycosylation,^[^ ^239^ ^]^ the authors noted that 2‐*O*‐acetyl and 2‐*N*‐acetyl containing glycosyl donors were ineffective as they may have led to interference of the reaction. Additionally, the cross‐coupling type glycosylation was limited to phenolic acceptors but not the wider range of saccharide based monol or polyol nucleophiles. Further advancement in this aspect would likely lead to a more versatile cross‐coupling type *O*‐glycosylation strategy.

In Ackermann's palladium catalyzed directed C─H activation, it is also noticeable at silyl‐protection (TIPS) of the glycal iodide was essential.^[^ ^240^ ^]^ Besides the limitation of solely employing monosaccharides as substrates, the directed C─H activation had not yet advanced to the stage where all carbon sites on a sugar could be precisely fucntionalized. The knowledge of distal directed C─H activation on saccharides is also nascent compared to that in the broader arene functionalization field. Further, Xu's 1,2‐*cis*‐aminoglycosylation^[^ ^241^ ^]^ and Qian's tandem borylation/*C*‐glycosylation^[^ ^242^ ^]^ necessitated fully protected glycals, and the former also required a Boc‐protecting group on the amination reagent. Polyol glycosyl acceptors were not employed in this work, and the field would greatly benefit from the addition of stereocontrolling elements to control site‐selectivity on the *O*‐nucleophile in this iron‐catalyzed strategy.

Zhang and Zhu's Au(I) catalyzed glycosylation using minimally protected glycosyl‐alkynyl‐napthoate stands out as a promising approach.^[^ ^243^ ^]^ However, the strategic minimally 6‐OH protection scheme mentioned earlier remained unresolved. Monosaccharide based glycosyl donors still predominates this protocol and further progress towards the employment of polysaccharides as electrophiles will expand the utility of this strategy. More importantly, it still remains to be seen if this approach could progress beyond carboxylic acid nucleophiles towards essential saccharide‐based nucleophiles required in oligosaccharide synthesis.

## 6 Summary and Outlook

This compilation summarizes transformative conceptual ideas in catalytic carbohydrate synthesis that are still very much a dynamic “works‐in‐progress” by diverse communities of researchers. As seen in this minireview, the categorization of these advances into fixed categories is non‐trivial due to the highly cross‐conceptual nature of the catalytic modes. This systemization is however a necessary entry point to facilitate broad understanding of more complex transformations that intertwines these concepts in real‐life examples. It is also pivotal to appreciate that excellent stereoselectivity outcome in carbohydrate synthesis is often a confluence of multiple concepts working in synergy.

Looking forward, the author believes that the traction gained in this emerging sphere is still confronted by grand challenges. One important question pertains to the conundrum of catalyst versus substrate control. A hard look at most reported cases revealed that there are no simple answers. While literature strongly establish the case for indisputable catalyst control, glycosyl substrate control as mentioned, is still a colossal factor of consideration.

Unquestionably, we are still far from the dream of universality and substrate‐agnostic strategies in catalyst‐controlled glycosylation. Further progress in site‐switchability strategies will definitely expand the reach of catalyst control in carbohydrate synthesis. Another big challenge is the systemic establishment of predictability of catalyst‐controlled site‐selective functionalization over all possible hydroxyl and C‐H sites in different variation of saccharides. While elaborate advances have certainly been made, catalyst‐controlled divergency has not reached the stage where a minimally protected or unprotected native saccharides can be precisely functionalized catalytically on a dictated functional group at every specified site. With the ever‐expanding toolbox of catalytic strategies, it is a foreseeable goal that the discovery of new catalytic routes into difficult to functionalize sites, such as the 2‐ or 4‐OH positions, will materialize. It is essential to appreciate that despite notable success, many of these newer methods had not yet been utilized in the total synthesis of oligosaccharides, natural products or glycoconjugates. While it is conceivable that current limitations of these methods may preclude their use for such endeavors, the author is confident that with on‐going strong advances in catalytic carbohydrate synthesis, newer catalytic strategies that offer potential solutions can be envisioned as the field continues to progress and mature. Eventually, it will be a common long‐term vision for both synthetic and carbohydrate chemists to discover an “ideal catalyst” or an “ideal catalyst family” that can predictably assimilate all possible glycosyl donors and acceptors in a selectivity‐switchable manner.

In light of the above challenges, we have further provided in this minireview critical evaluations as separate sections at the end of each of the depicted categories to assist newer synthetic researchers in this emerging field. This will be beneficial for organic chemists to better appreciate the intricacies behind glycosyl substrate design, protecting group schemes and catalyst structures in light of new catalytic progress.

Notwithstanding, the author believes there are good reasons for broad optimism as some of this newly emerging chemistry had found useful applications in varied biologically relevant settings. The phenanthroline catalysis developed by Nguyen was demonstrated in the octasaccharide synthesis of an α‐glucan adjuvant.^[^ ^100^ ^]^ Niu's merging of photoredox catalysis and noncovalent stereocontrol enabled the access of a considerable series of native oligosaccharide sequences.^[^ ^177^ ^]^ The Niu group had also reported other instances of glycoconjugations using photocatalyzed techniques.^[^ ^244^, ^245^ ^]^ Further, Koh, and Davis showcased the use of photocatalyzed *C*‐glycosylations in post‐translational glycosylation of proteins.^[^ ^185^ ^]^ In our seminal σ‐hole based glycosylation strategy using XB catalysis, the new method development facilitated the phenotypic‐screening identification of novel non‐natural glycosides with potential applications against acquired cancer resistance.^[^ ^134^ ^]^ A lately appearing report further showed the amenability of mild XB catalysis in the total synthesis of a nucleoside antibiotic, cytosamine.^[^ ^246^ ^]^

To end off, the author hopes that our depiction of this infant field would offer the broad synthetic readership a stepping stone into the rapidly expanding sphere of catalytic carbohydrate synthesis, and to inspire ground‐breaking ideas in this fascinating domain. Ultimately, the converging trajectory of the carbohydrate chemistry and frontier catalytic domains is a heartening synthetic development to be embraced. It promises plentiful opportunities for discovery of new catalytic phenomena and far‐reaching solutions to long‐standing selectivity problems across the broad spectra of chemical sciences.

## Conflict of Interests

The author declares no conflict of interest.

## Data Availability

The data that support the findings of this study are available from the corresponding author upon reasonable request.
